# An assessment of ocean thermal energy conversion resources and climate change mitigation potential

**DOI:** 10.1007/s10584-025-03933-4

**Published:** 2025-05-12

**Authors:** Anna G. Nickoloff, Sophia T. Olim, Michael Eby, Andrew J. Weaver

**Affiliations:** School of Earth and Ocean Sciences, University of Victoria, PO Box 1700, Victoria, BC V8 W 2Y2 Canada

**Keywords:** Ocean Thermal Energy Conversion (OTEC), Renewable energy systems, Earth system modelling, Climate change mitigation, Resource assessment

## Abstract

Ocean thermal energy conversion (OTEC) is a renewable energy system that harnesses the thermal gradient between surface and deep waters. Many multi-century simulations with a fully coupled climate-carbon cycle model are presented to explore the amount of extractable energy and the climate change mitigation potential from the widespread implementation of OTEC. The sustainability of OTEC power generation was assessed for present and possible future climate states. A warmer climate reduced the sustainable power potential of OTEC. OTEC could briefly produce over 35 TW of power and, depending on the climate state, maximum power production rates of 5 to 10 TW were found to be sustainable on multi-millennial timescales. Over 500 years of simulation, with a high emission scenario (equivalent to RCP8.5), the power from OTEC deployments, with peak power generation ranging from 3 to 15 TW at the year 2100, resulted in cumulative emission reductions equivalent to 36% to 111% of historical carbon emissions from 1750 to 2023 relative to the scenario without OTEC. Such significant emissions reductions coupled with sustained OTEC-induced mixing led to globally averaged atmosphere temperature decreases of up to 2.5 ºC by the year 2100 and up to 4 ºC by the year 2500 compared to a scenario without OTEC. While caution is required, and the engineering challenges would be large, early indications suggest that the large-scale implementation of OTEC could make a substantial contribution to climate change mitigation.

## Introduction

Global energy demands are rising exponentially due to rapid population growth and increased technological advances (Olabi and Abdelkareem 2022). Fossil fuels provide most of the world’s energy despite their well-documented detrimental environmental impacts (Curtin et al.^2019^; Yang et al. 2021). In the year 2021, global power generation was estimated to be 20 TW and about 79% of this power was derived from fossil fuels, resulting in emissions of roughly 10 Pg of carbon per year (IEA 2021). Some of this demand could be met more efficiently with a transition to primary renewable energy sources which would support humanity’s increasing energy needs while maintaining the global climate system.

Ocean Thermal Energy Conversion (OTEC) is a form of marine renewable energy that harnesses the thermal gradient between warm surface water and cool deep ocean water (DOW) to power a heat engine and produce useful work. Since its conception in 1881, OTEC has sparked much research and debate around the extent to which it could be a viable technology for power generation (D’Arsonval 1881; Lennard 1995; Dubois et al. 2008; Bernardoni et al. 2019; Herrera et al. 2022). Only two land-based OTEC plants are currently operational: a 50 kW double Rankine system at Saga University, Japan, for demonstrations and model validations, and a 105 kW closed-cycle system at Hawaii's Natural Energy Laboratory, powering about 120 homes (Martin et al. 2016; Makai Ocean Engineering 2018).

**Fig. 1:**
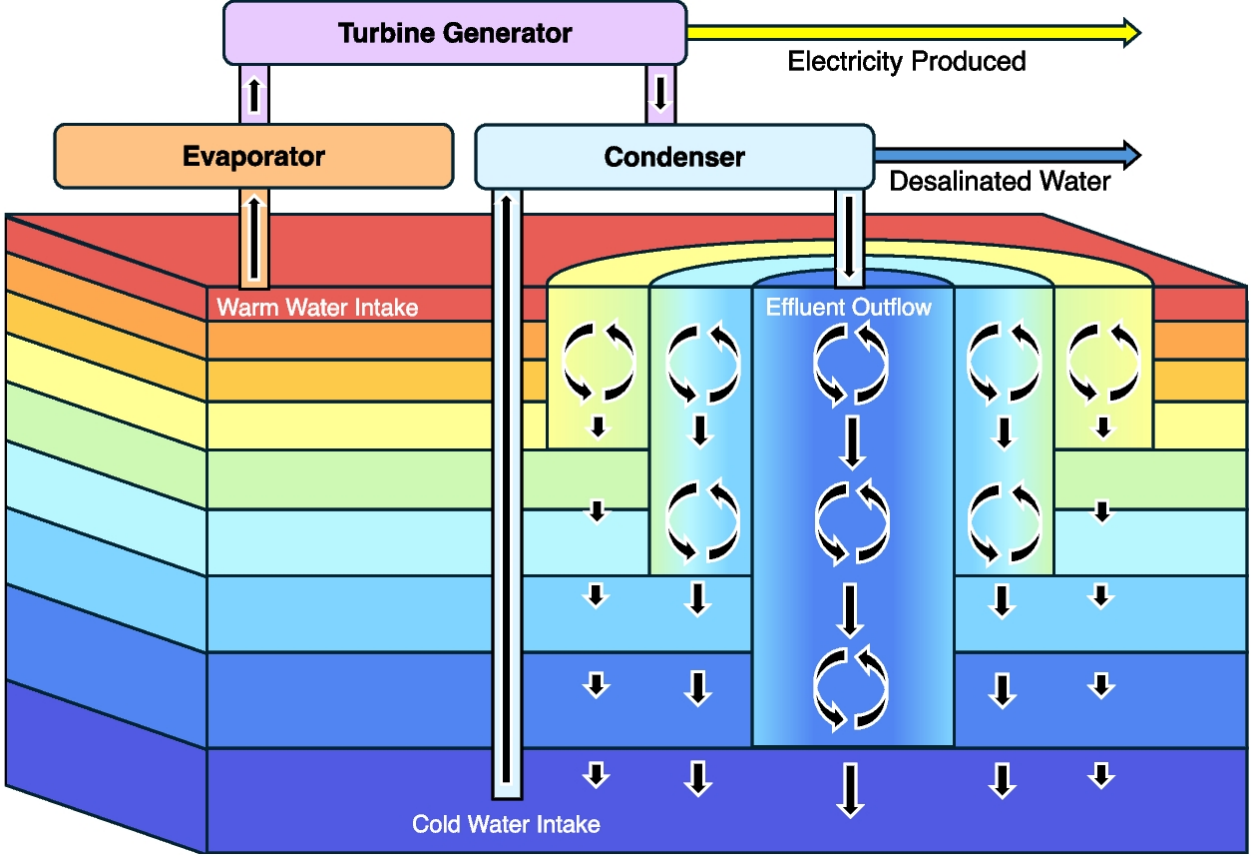
Simplified schematic diagram of an open-cycle OTEC system with a simplified representation of the vertical mixing of the effluent entrainment plume. Colours denote seawater density with warm colours representing low densities and cool colours representing high densities. Black vertical arrows represent advective velocities and curved black arrows represent convective mixing. The effluent mixing scheme shown here does not attempt to represent the horizontal distribution of mixing but only to improve how the vertical mixing varies with the amount of entrainment. This diagram is stylized and not drawn to scale

Figure 1 shows a simplified representation of the OTEC power generation process. The system requires the use of a working fluid which can either be the seawater itself in open-cycle systems or another fluid with a low boiling point, commonly anhydrous ammonia, in closed-cycle systems. In both open and closed systems, the working fluid is flash-evaporated in a vacuum chamber, using heat from warm surface waters, which creates a low-power system that drives a turbine generator (Wang et al. 2008). The cool DOW is used to condense the working fluid steam after it passes through the turbine (Vega 1995). In open systems, where water is used as the working fluid, the cycle produces desalinated water as a by-product (Vega 1995). The production of desalinated water is immensely valuable in regions that currently rely on fossil fuels to desalinate drinking water, particularly diesel-reliant small island districts (Parker et al. 2023).

OTEC power generation relies solely on the presence of a temperature differential between the sea surface and depth. Unlike other intermittent forms of renewable energy (wind or solar), OTEC allows for continuous power generation. Thus, OTEC has the potential to contribute significantly to baseload energy (Assareh and Dejdar 2022). OTEC power generation is most efficient with large shallow-to-deep seawater temperature gradients. Previous studies assumed OTEC requires temperature gradients upwards of approximately 18ºC to generate power efficiently (Nihous 2007a,b; Rajagopalan and Nihous 2013a,c). Locations with a sufficient temperature gradient to support OTEC implementation are largely constrained to deep warm seas in the tropics. OTEC relies on smaller temperature gradients than similar forms of heat engine technology, necessitating large volumes of warm surface and cool deep water to generate significant quantities of electricity, posing a significant implementation challenge (Rajagopalan and Nihous 2013c). Other challenges include the OTEC’s low thermodynamic efficiency of approximately 3%, the necessity to pump immense amounts of water to produce a meaningful amount of energy (2.5—3 m^3^/s per MW of net power), and significant costs (Nihous 2005; Chung and Wu 2024).

While OTEC could provide ample amounts of continuously available renewable energy, the economic feasibility of the technology is still under question (Bernardoni et al. 2019; Langer et al. 2020, 2022; Giostri et al. 2021). OTEC has a Levelized Cost of Energy (LCOE) of 0.145—0.63 USD_2023_/kWh which is relatively high when compared to that of other forms of renewable energy like solar photovoltaic and onshore wind systems that have LCOE values of 0.118 USD_2023_/kWh and 0.0714 USD_2023_/kWh, respectively (Shen et al. 2020). Therefore, a large-scale adoption of OTEC as discussed in this paper would likely require significant subsidies. However, the LCOE of OTEC could decrease with revenues from the production of desalinated water and emissions reductions when transitioning from fossil fuels to renewables (Giostri et al. 2021), with potential additional financial benefits through carbon tax initiatives. Unlike other renewables, OTEC can provide higher-valued baseload energy which could further lower generation costs. OTEC platforms could also directly supply energy for shipping, remote high-intensity computing, or direct CO_2_ capture and storage (Olim et al. 2025).

While the economic feasibility of deploying OTEC on a massive scale is beyond the scope of this study, it should be noted that any deployment that would make a substantial contribution to climate change mitigation would present enormous economic and engineering challenges and may come with some environmental costs (Nickoloff et al. Under Review). These obstacles have prevented the technology from being economically and environmentally viable, although interest in OTEC has continued due to the increasing need for fossil fuel alternatives and a growing global energy budget. While early theoretical estimates suggested OTEC power potentials as high as 1000 TW, more recent computer model estimates of sustainable OTEC power levels still vary significantly, from as much as 14 TW (Rajagopalan and Nihous 2013b) to as little as 3 TW (Nihous 2005, 2007a). The discrepancy in power estimates arises from the uncertainty surrounding the limiting factors for reservoir renewal. The larger early and more theoretical estimates only considered factors like insolation and evaporation rates (Vega 1995; Masutani and Takahashi 2000), whereas the more recent estimates consider factors like rates of DOW formation, environmental safety, and modifications to the ocean thermal structure (Zener 1973; Penney and Daniel 1989; Johnson 1992; Avery and Wu 1994; Nihous 2018; Rau and Baird 2018).

In several studies (Nihous 2005, 2007a, b), modelling of a one-dimensional oceanic water column was conducted to investigate the effects of OTEC on the thermal structure of the ocean. Their analyses provided estimates of the OTEC reservoir size ranging from 3 to 5 TW (Nihous 2005, 2007a). The first three-dimensional study on OTEC resources using an oceanic general circulation model was by Rajagopalan and Nihous (2013a). The study used the Massachusetts Institute of Technology General Circulation Model (MITgcm) which was relatively coarse (4° × 4°), had an uncoupled ocean–atmosphere system and lacked sea ice or carbon model components. It was found that OTEC could sustain maximum power production rates of 14 TW and power generation on the order of 7 TW did not result in substantial perturbations of ocean temperatures. In the same year, Rajagopalan and Nihous published a similar study (2013c) using the MITgcm with a resolution of 1° × 1°. This study broadly confirmed their earlier findings. Jia et al. (2018) employed the MITgcm to study OTEC resources with the inclusion of simple atmospheric feedback. They found the OTEC power resource to be between 8 to 10.2 TW globally. OTEC mixing processes in all of these previous studies (Nihous 2005, 2007a,b; Rajagopalan and Nihous 2013a,b,c) were parameterized as sinks of cool or warm water at intake depths and as a source of mixed intake water (effluent outflow) as a source at the depth of neutral buoyancy. This scheme appears to ignore entrainment, which would result in an overestimation of effluent penetration and neglect any reduction in stratification due to mixing between these sink and source depths.

This study builds on previous research by incorporating a fully coupled climate-carbon cycle model to investigate the sustainability of the OTEC power resource and OTEC's potential for climate mitigation. The experiments presented have several novel aspects. A new sub-grid parameterization of OTEC mixing and an adaptive OTEC plant deployment scheme are presented. A more quantitative estimate of the amount of"sustainable"OTEC power generation is found and its dependence on the climate state is demonstrated. This is also the first study to assess the climate mitigation potential for various future OTEC deployment scenarios which contributes to our understanding of OTEC's potential role in a sustainable energy transition.

## Methods

### Climate model description

This research utilized version 2.9 of the University of Victori Earth System Climate Model (UVic ESCM). The UVic ESCM (Weaver et al. 2001), is a coupled, global model of intermediate complexity, implemented in Fortran 90. It operates on a zonal and meridional spherical grid with resolutions of 3.6º and 1.8º, respectively. The atmospheric model is a single-layer energy-moisture balance model with parameterized dynamic feedbacks. The sea ice model includes simple two-level thermodynamics (Hibler 1979; Bitz and Lipscomb 1999) and dynamics utilizing an elastic viscous plastic ice rheology (Hunke and Dukowicz 1997). Snow cover is approximated with a single-height layer. The ocean model is a 3-D primitive equation oceanic general circulation model (Pacanowski 1995) with 19 vertical layers that increase in thickness parabolically from 50 m at the surface to 518 m at depth. Vertical diffusivity varies with depth according to Bryan and Lewis (1979) and mesoscale-eddy mixing is parameterized according to the scheme of Gent and McWilliams (1990). Ocean biology is represented by three plankton classes: diazotrophs, which can fix nitrogen, other phytoplankton, and zooplankton (Schmittner et al. 2008). Two macronutrients are modelled: nitrate and phosphate. The model simulates both oxygen depletion and denitrification. The dynamic vegetation model represents the biosphere soil carbon and five unique plant functional types: broadleaf tree, needleleaf tree, C3 grass, C4 grass, and shrub (Meissner et al. 2003). The land surface scheme consists of a simplified version of the scheme described by Cox et al. (1999) and uses a single soil layer to represent temperature and moisture content (Meissner et al. 2003).

### OTEC model description

OTEC plants in this study are assumed to be autonomous floating platforms and any excess energy produced by a plant is converted to some energetic chemical form, such as hydrogen or ammonia, and shipped to land. It is also assumed that plants are not constrained to be near land. Uptake and discharge flows are modelled as sub-grid-scale mixing processes before being spread over the larger-scale ocean model grid cell. Cold water is drawn from the bottom of a large diameter pipe at the specified depth of uptake (about 1100 m) and mixed with an appropriate volume of warm surface uptake water. This mixture is discharged at the specified discharge depth (about 20 m). Discharge at lower depths may reduce local biological impacts in the photic layer (~ 100 m deep). The model is not sensitive to changing the discharge depth over the first 130 m (first 2 ocean model layers), although this may just be a limitation of the coarse vertical resolution. The mixed discharge is denser than the surface water and is mixed downward (using the course resolution model’s complete convection scheme) to a depth of neutral buoyancy. As the plume descends it entrains the surrounding water which lowers the plume’s density and limits its depth of penetration. Entrainment is parameterized over a circular area (500 m radius) and discretized as ten vertical columns. The surface area of each column represents the areas of ten sandwiched, 50 m thick concentric rings from the centre to the edge of the entrainment area (see Fig. 1). The radius of entrainment is specified to be similar to the near-field radius of horizontal mixing found in Rocheleau and Grandelli (2011) and Rodríguez Buño (2013). At each ocean model timestep, the initial depth profile for each column is set to be the same as in the course resolution model and the discharge volume is distributed linearly from a maximum value at the centre of this area of entrainment to zero at the outer edge. This results in the discharge volume per unit area, and thus the depth of penetration of discharge water, decreasing away from the point of discharge. The outermost ring represents the portion of the discharge that has the most entrainment of surrounding water and thus the lowest amount of vertical penetration of effluent, while the innermost circle represents the portion of the plume with the least entrainment and the deepest penetration. Vertical advection and convection are calculated for each of the ten columns separately. The vertical advective velocities are found from the discharge volume divided by the horizontal area for each column to ensure mass conservation. These columns are then mixed laterally at all depths, weighted by the surface area of the columns (scaled up for all plants in a grid cell), relative to the surface area of the rest of the ocean model grid cell.

In reality, dense discharge plumes tend to spread out horizontally, entraining additional water, as they descend. Sub-grid entrainment is difficult to parameterize accurately. Given that only changes in the vertical structure of the water column affect the large-scale model, this parameterization of entrainment does not attempt to simulate the horizontal distribution of mixing in the effluent plume but represents the decreased vertical mixing with increased entrainment. It may be that this scheme under, or over-estimates the vertical mixing in portions of the water column, but this simple parameterization can capture the first-order effect of reduced effluent penetration due to entrainment. The course resolution ocean model was not very sensitive to the discretization of entrainment, but it was moderately sensitive to large changes in the radius of entrainment. If the radius is reduced, the vertical penetration of effluent is increased and if the radius is increased, penetration is decreased. This novel effluent plume parameterization is a first attempt at finding the middle ground between releasing OTEC effluent as a point source at the level of neutral buoyancy (as in Nihous 2005, 2007a,b; Rajagopalan and Nihous 2013a,b,c; Jia et al. 2018), which would overestimate the depth of effluent penetration, and releasing OTEC effluent over the area of the entire coarse-grid cell surface (similar to the artificial upwelling scheme in Keller et al. 2014), which would underestimate the vertical mixing of deep-water discharge. Although the simple entrainment parameterization used here is arguably better than previous schemes, future refinements may improve our estimates of OTEC-induced vertical mixing.

OTEC plants are assumed to be closed cycle and the power produced by a single plant is approximated by Eq. 1 (see Rajagopalan & Nihous (2013b) for more details).

1 $${P}_{net}={\omega }_{cw} \rho {c}_{p} {\varepsilon }_{tg} \left(\frac{9}{80}\frac{\Delta {T}^{2}}{T}-\frac{9}{200}\right)$$

Here ${\omega }_{cw}$ represents the volume flow rate of OTEC deep seawater (m^3 ^s^−1^), $\rho$ is the mean seawater.

density (1,025 kg m^−3^), ${c}_{p}$ is the specific heat (4,000 J kg^−1^ K^−1^), ${\varepsilon }_{tg}$ is the turbo-generator efficiency (0.75), $T$ is the intake seawater temperature, and $\Delta T$ is the temperature difference between surface and deep seawater intakes. The numerical coefficients account for a flow rate ratio of 1.5 of surface-to-deep seawater, and seawater pumping power losses equal to 30% of the turbo-generator output at standard conditions.

For an OTEC plant to produce about 100 MW in “average” tropical water column temperatures, a volume flow rate of 314 m^3 s−1^ is specified for each plant. Given the volume flow rate is proportional to the cross-sectional area and the velocity of the flow, this volume flow rate could be generated by adjusting the diameter or number of pipes, or the flow velocity. For example, a single 10 m diameter pipe with a flow velocity of 4 m s^−1^, or two pipes of 10 m diameter and a flow velocity of 2 m s^−1^, would produce a volume flow rate of 314 m^3 s−1^.

These parameters have been defined somewhat arbitrarily and will not necessarily reflect ocean conditions and plant configurations in potential future OTEC deployments. Rather, these variables were used as a reasonable estimate of potential site conditions and are consistent with previous studies on the topic (Rajagopalan and Nihous 2013b; Jia et al. 2018). The aim is to have plants produce power in the range of 100 MW. Using Eq. 1, OTEC plants in tropical regions (31.5 ºN to 31.5 ºS) produce a mean power output per plant of 106.5 MW with year 2000 thermal gradients. Globally, year 2000 potential power production rates for a single OTEC plant ranged from 222.5 MW in the Equatorial Pacific to sub-zero values at high latitudes. Negative power values represent areas where operational power plants would consume more power than could be produced.

The main constraint on the energy derived from a given grid cell is the temperature gradient between waters at the ocean surface and those at the cold-water uptake depth. An annual average of the shallow-to-deep temperature gradient, updated every 5 days for the previous 365, is used to calculate the running annual average potential power from Eq. 1. The number of OTEC plants built is a function of the OTEC power goal in each scenario (which can vary with time as production ramps up) and the annual average power that plants produce in each grid cell. Therefore, as thermal gradients become depleted, and power generation becomes less efficient, net power output per plant is reduced and a greater number of OTEC plants are required to reach an OTEC power goal. If the power level generated by a plant falls below a set minimum, it is shut down and added to the number of plants available for deployment.

Every 5 days, the potential annual average energy production in each ocean grid cell is reassessed and sorted. Any newly built or redeployed plants are placed one at a time in grid cell, following the ranking of energy production potential, given the constraints that the grid cell meets the minimum power and area requirements. The next available plant is deployed in the grid cell with the next highest power potential until all of the plants are placed. If the number of plants to be placed is greater than the number of grid cells that meet the minimum power and area requirements, deployment is started again with the grid cell with the largest potential power production. This is continued until all available plants are placed or there are no areas left that meet the minimum power and area requirements. This means that more than one plant may be placed in a grid cell over a single deployment period. Any available plants that cannot be placed during this deployment are added to the total available for future placement. The 5-day energy reassessment is the maximum possible rate of deployment and plants are only deployed if and when they are available. To minimize plant redeployment costs, existing plants are not redeployed unless they have been shut down for falling below the set minimum power level.

To ensure that the modelled OTEC deployment is reasonable, OTEC plant design parameters were specified based on projected building designs from recent literature (Rajagopalan and Nihous 2013b; Ma et al. 2023; Habib et al. 2023). A sensitivity analysis of the model to OTEC design parameters, such as the depth of OTEC pipes, volume flow rates, depth, and area of discharge, was performed. Although varying the discharge plume radius influenced the depth of OTEC-related convective mixing and the number of plants required to meet the specified OTEC power output, the model was not highly sensitive to changes in other parameters.

### Experimental design

The initial conditions for all model experiments were derived from a long 10,000-year equilibrium model spin-up at the year 850 CE with only seasonally varying forcing. The spin-up was integrated through to the year 2000 with transient historical forcings specified by the Climate Model Intercomparison Project Five (CMIP5) which was developed for the IPCC’s fifth assessment report (IPCC 2014). This formed the initial condition for all subsequent experiments. From 2000 to 2300, extended Representative Concentration Pathway (RCP) forcings were specified and beyond 2300, year 2300 forcing was specified with only seasonal variations (Zickfeld et al. 2013). CO_2_ concentrations are specified for RCP scenarios. A control simulation (without OTEC deployment) was performed to allow the UVic ESCM to diagnose CO_2_ emissions consistent with specified CO_2_ concentrations and other forcing until the end of the modelled period (year 2500). All subsequent experiments were driven by these diagnosed CO_2_ emissions rather than concentrations.

#### OTEC power resource

To reduce the uncertainty in OTEC power potential, a series of idealized experiments were carried out using two bounding future climate scenarios. For these experiments, requested OTEC power levels were immediately set to various maximum power goals in the initial year. OTEC deployment was not constrained by the number of plants built or the area available and the minimum power requirement was set to zero. Maximum power goals ranged from 3 to 35 TW in 1 TW increments. Climate forcing, including CO_2_, was specified throughout the simulations so OTEC does not affect the atmospheric concentration. The first series of experiments started in the year 2000 and continued with seasonally varying, year 2000 forcing. In the second series of experiments, the model was integrated without OTEC deployment from the year 2000 to 2300 using RCP8.5 climate change forcing. Thereafter, OTEC power production was initiated, and the simulations continued with seasonally varying year 2300 forcing. These two experiments represent two extremes of potential future climate states. One, with relatively little change from the year 2000 and the other, being the most extreme RCP scenario. Reality is likely to fall between these extremes.

#### OTEC climate change mitigation

To assess the potential of OTEC for future climate change mitigation, scenarios with varied OTEC power output goals were generated with RCP8.5 climate forcing, beginning in the year 2000 and terminating in 2500. Before resolving to use RCP8.5 as the default future forcing, the significance of the RCP scenario on the model output was considered. The pathway chosen impacts the resulting atmospheric CO_2_ levels over time. This will have wide-reaching effects on global temperatures, ocean chemistry, and even the amount of OTEC power available. RCP8.5 was selected to be the most appropriate pathway as unlike the other pathways (2.6, 4.5, and 6.0), RCP8.5 accounts for neither the employment of renewable technologies nor strategies for reducing greenhouse gas emissions. As the levels of OTEC deployment discussed in this paper would represent a massive movement towards green technologies, it is essential to use an emission scenario that does not already account for the type of emission reductions attributed to any OTEC implementation. Therefore, diagnosed emissions from RCP8.5 are solely used in these experiments.

Given that RCP 8.5 is a high emissions scenario, all diagnosed emissions are assumed to be anthropogenic and are considered to be mitigable by a green energy source like OTEC. When converted to equivalent electrical energy, these emissions are referred to as the climate mitigation power demand. The model reduces the diagnosed RCP emissions by the equivalent amount of fossil-fuel emissions displaced by OTEC-power production. The emission reduction from OTEC deployment was approximated as 1.5 Pg per year of carbon for every 1 TW of electricity produced. This ratio was roughly based on current estimates of the carbon intensity of all electrical power production (Moore et al. 2022) and assuming that only 60% of electricity is derived from fossil fuels (Ritchie et al. 2017). The calculated emission reduction was then subtracted from the specified carbon emissions scenario.

Suitable OTEC locations are defined as grid cells with adequate shallow-to-deep temperature gradients and enough area to support OTEC power generation. Each OTEC plant required a set minimum area (200 square kilometres) to avoid overcrowding of plants. A minimum area of 200 square kilometres would result in a minimum average distance between plants of about 16 km ($2 \sqrt{(200/\pi }$).

OTEC placement was restricted to areas with power potentials greater than 75 MW, which corresponds roughly to the 18 ºC threshold temperature used in other modelling efforts (Nihous 2007a,b; Rajagopalan and Nihous 2013a,b,c). Plants were decommissioned and not relocated if one of three conditions was true: (1) OTEC production goal is met, (2) climate mitigation power demand is met, or (3) no suitable OTEC locations remain. The climate mitigation power demand parameter limits OTEC power production to the power that would have otherwise been sourced from fossil fuels given a specific emissions scenario.

OTEC power generation commenced in 2030 and increased according to a raised negative cosine function (a parameterization of an increasing plant production) to reach the OTEC maximum power production goal by the year 2100. Experiments were produced for OTEC maximum power goals of 3, 5, 7, 10, and 15 TW which will be referred to as *OTEC3, OTEC5, OTEC7, OTEC10,* and *OTEC15,* respectively. Additionally, a control simulation (*OTEC0*) was generated in which global warming occurs under RCP8.5 forcing without OTEC power generation. These OTEC power production rates range from the more conservative estimates of OTEC power limits to more optimistic estimates while remaining within previous estimates for OTEC power production as limited by environmental safety (Avery and Wu 1994).

## Results and discussion

### OTEC power resource

**Fig. 2:**
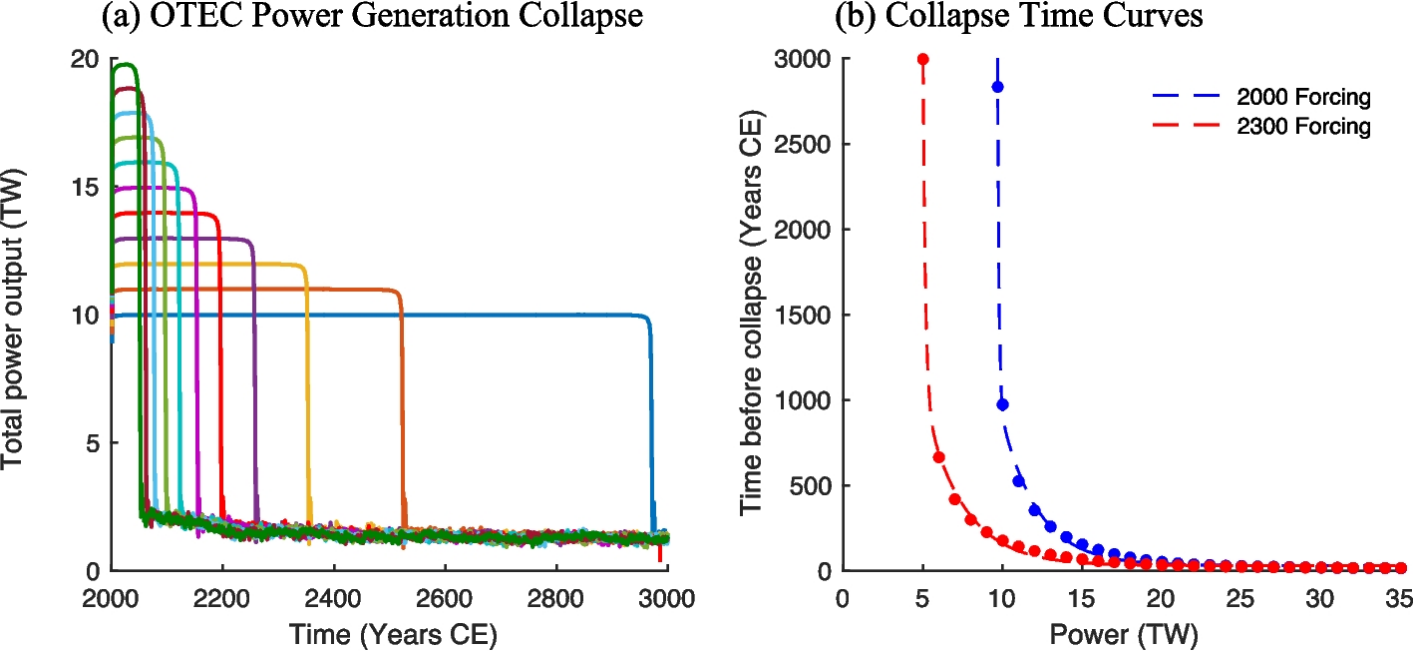
(**a**) Total power output in terawatts (TW) for simulations ranging in power from 10 to 20 TW with constant year 2000 forcing. Scenarios with greater power outputs are omitted for clarity. Rapid reductions to power outputs mark power generation collapse. (**b**) Relationship between the amount of power drawn via OTEC and the years before the system collapses. Blue dots represent data derived from each modelled simulation. The dashed red line represents an offset double negative exponential curve fitted to the data described in Eq. 2

Estimations of the potential energy capacity of OTEC have ranged anywhere from 3 to 1,000 TW (Zener 1973; Penney and Daniel 1989; Johnson 1992; Avery and Wu 1994; Vega 1995; Masutani and Takahashi 2000; Nihous 2005, 2007a, 2018; Rajagopalan and Nihous 2013a,c; Jia et al. 2018; Rau and Baird 2018). One goal of this study is to investigate this question with a more comprehensive earth system model to reduce this uncertainty. To understand the relationship between power drawn and the length of time OTEC power can be extracted, two ensembles of model simulations were conducted as outlined in Sect. 2.3.1. In these simulations, power production goals varied from 3 to 35 TW and climate forcing, including CO_2_ concentrations, were held fixed at either year 2000 or year 2300 levels. Given the adaptive deployment scheme described in Sect. 2.2 and the unlimited number of plants available for immediate deployment, the initial plant deployment is relatively uniform over all areas capable of generating power. As gradients become depleted and plants are relocated to maximize energy production, they become concentrated in the Indo-Pacific Warm Pool. The power production goal was set at the initial year and held constant until it could no longer be sustained by the ocean system and power generation collapsed. The collapse of OTEC power generation was defined to occur when power output fell from the output goal to values below 2 TW (Fig. 2). The length of time that the ocean could sustain each power level with year 2000 forcing (Eq. 2) and year 2300 RCP8.5 forcing (Eq. 3) was found to follow offset double negative exponential relationships both with r-squared values of 0.999.

2 $${T}_{c}=1.86\times {10}^{12}\times {e}^{-11.7({P}_{sp}-7.91)} +2.10\times {10}^{3}\times {e}^{-0.434({P}_{sp}-7.91)}-31.0$$

3 $${T}_{c}=1.18\times {10}^{6}\times {e}^{-6.42({P}_{sp}-4.00)} +1.47\times {10}^{3}\times {e}^{-0.405({P}_{sp}-4.00)}-31.1$$

Here ${T}_{c}$ is the time in years after OTEC commencement that a given goal power level can be sustained before collapse and ${P}_{sp}$ is the specified power level in TW.

Power generation collapses are sudden because there is no constraint on the number of plants being deployed in order to reach a power goal. As power production is reduced, due to the depletion of temperature gradients from OTEC mixing, more plants are deployed until the goal is met. Once every viable location becomes depleted, the ocean is no longer able to provide the energy required, and power generation quickly collapses.

The amount of time that a given power level could be sustained was highly sensitive to the selected forcing scenario. Simulations that used year 2000 forcings were able to support power generation at all levels for significantly longer than those that used year 2300 forcings (Fig. 2). This is in direct contradiction to previous work which predicted that global warming would augment OTEC resources (Du et al. 2022). The discrepancy likely arises from the disparate timescales on which the surface and deep oceans warm. On decadal timescales like the ones considered by Du et al (2022), additional anthropogenic atmospheric CO_2_ warms the sea surface while deeper waters are relatively unchanged and therefore ocean thermal gradients increase. On longer timescales, deep water formation is affected, and intermediate and bottom waters are warmer, leading to diminished surface-to-deep thermal gradients. While OTEC-induced mixing does contribute to the eventual reduction in tropical thermal gradients, the change is mostly due to global warming. In *OTEC0*, where no OTEC-induced mixing occurs, thermal gradients degraded over time and global average thermal gradients between 0 and 1000 m depth were 1.5 ºC less than year 2000 values by 2500. In *OTEC10*, year- 2500 thermal gradients were reduced by an additional 0.3 ºC, indicating that roughly 20% of gradient reduction was caused by OTEC-induced mixing and the remaining 80% was caused by global warming.

Under year 2000 forcing, the ocean sustained a power generation rate of 10 TW for nearly 1000 years whereas the system could sustain 15 TW for roughly 150 years and 20 TW for roughly 50 years (Fig. 2). Notably, the modelled scenarios with OTEC power generation rates of less than 9.7 TW did not experience collapse over a 10,000-year simulation. In contrast, simulations that used year 2300 forcing sustained the generation of 10 TW for roughly 180 years, 15 TW for roughly 70 years and 20 TW for 35 years. In these simulations, power generation rates under 5 TW were sustainable over the entire 10,000-year modelled period. This suggests the amount of sustainably extractable energy via OTEC processes ranges from 5 to 10 TW, depending on the state of the climate. After the collapse of power generation, power output in all simulations converged to a year 3000 mean of 1.3 TW with little variation in power output between different climate states or peak power outputs.

This relationship suggests that, while the ocean system may briefly be able to produce more than 35 TW of power, this level of power generation cannot be sustained. The more power derived from the ocean system; the sooner power generation collapses down to about 1 TW. Power generation rates of less than 5 to 10 TW (depending on the climate state) were sustainable over multi-millennial timescales and rates of 1.3 TW and below would presumably be permanently stable. At power generation rates above this, energy cannot be derived from the system indefinitely without the threat of collapse. Power can be drawn at lower levels to extend the period where significant amounts of energy can be produced.

### OTEC climate change mitigation

**Fig. 3:**
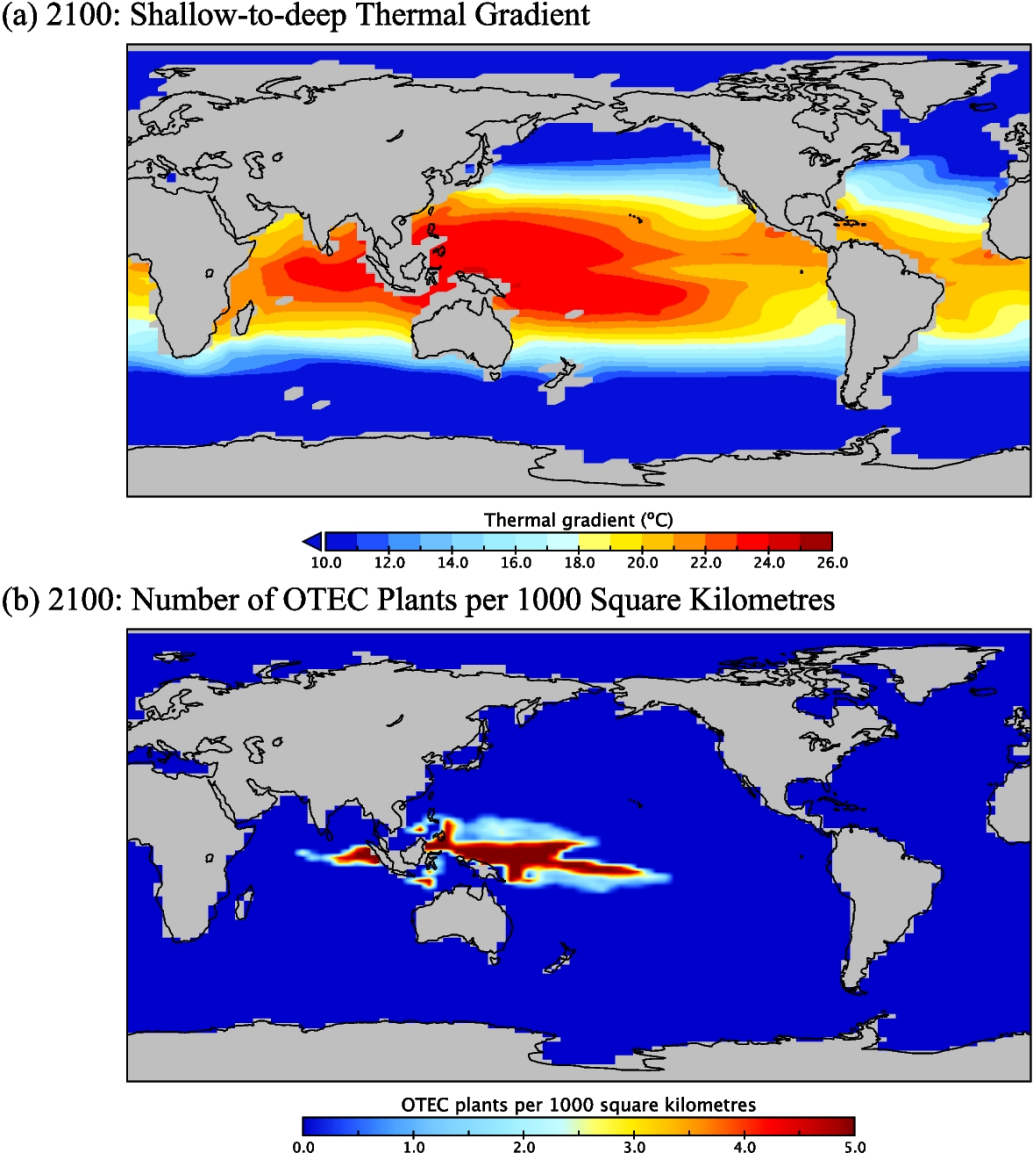
(**a**) Temperature gradients in ºC between 0 and 1000 m depth in 2100 for the 10 TW OTEC deployment and the RCP8.5 scenario. Regions in warm shades (red, orange, yellow) denote areas with a sufficient depth and temperature gradient to potentially support OTEC. (**b**) Density of OTEC plants at peak power production in the year 2100 for a 10 TW OTEC deployment and the RCP8.5 scenario. Both panels show global distributions. The OTEC plants in panel 3b are almost exclusively deployed in the Indo-Pacific warm pool since this area minimizes the number of plants required to produce 10 TW of electricity by the year 2100

Another goal of this study is to investigate the climate mitigation potential of OTEC, given the various power goals outlined in Sect. 2.3.2. After the commencement of OTEC in 2030, power generation in the *OTEC3, OTEC5, OTEC7, OTEC10,* and *OTEC15* scenarios increased smoothly to reach maximum power levels by 2100. Following this, specified power production was sustained until generation became limited by declining climate mitigation power demands or a lack of suitable locations for OTEC. Plant placement was greatest in the West Pacific and parts of the Indian Ocean due to the large thermal gradients present in these regions (Fig. 3a). Since deployment in grid cells with large temperature gradients was prioritized, OTEC plant locations were highly concentrated with densities of up to five plants per thousand square kilometres. As more OTEC plants were added and thermal gradients in areas with OTEC deployment became gradually depleted, plant placement became more dispersed and spread eastwards in the Pacific Ocean, westwards in the Indian Ocean, and poleward. By 2100, OTEC placement extended from 67 ºE to 211 ºE and from 15 ºN to 15 ºS (Fig. 3b).

**Fig. 4:**
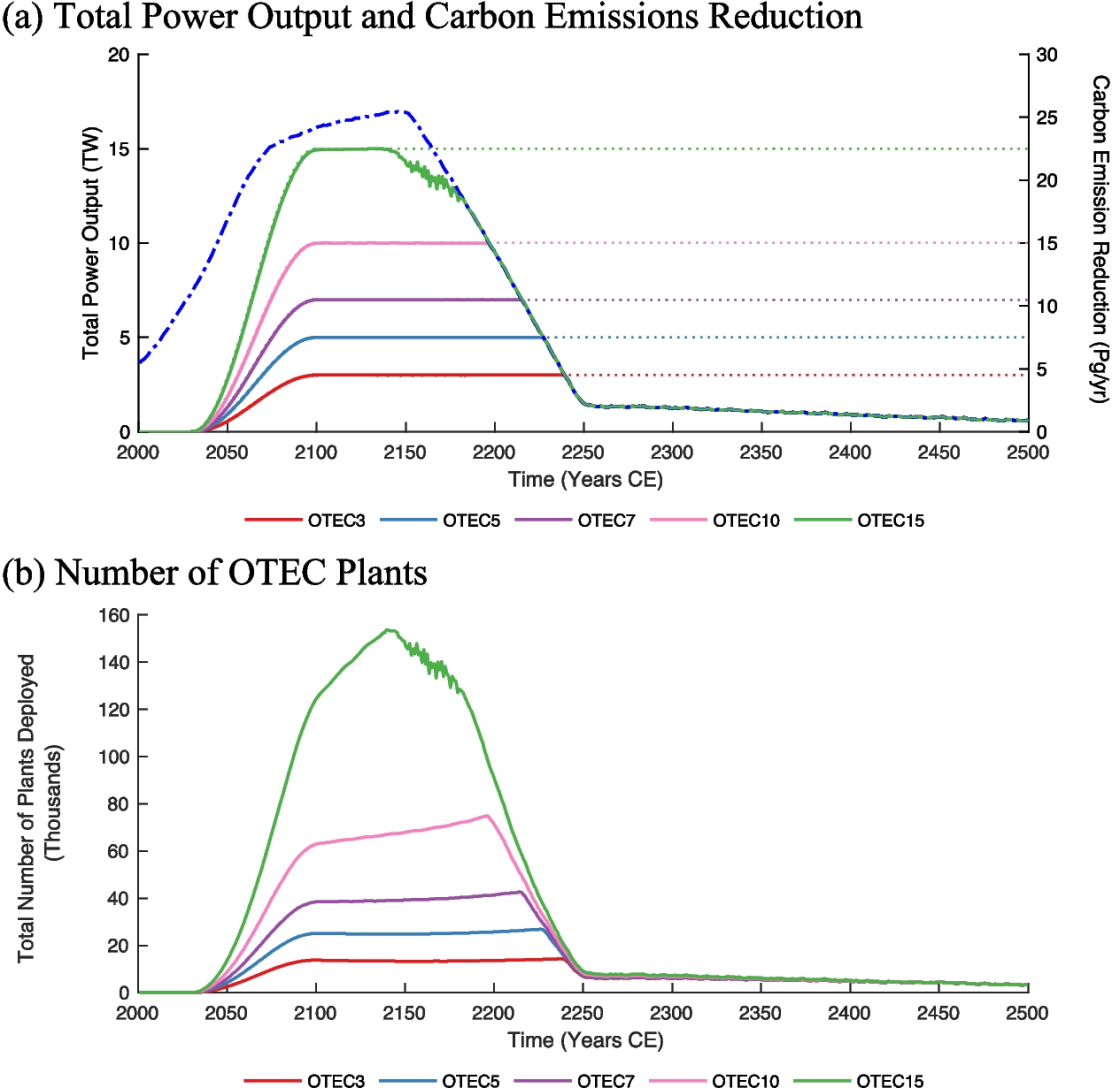
(**a**) Total OTEC power output in terawatts (TW) on the left axis and OTEC-associated carbon emission reductions in Pg/yr of C on the right axis from 2000 to 2500. The solid lines denote the net power output from OTEC (left axis) and the corresponding emissions reductions (right axis) for each simulation. The dotted lines mark the power goal, and the blue dashed line represents the climate mitigation power demand limitation. (**b**) Total number of OTEC plants deployed from 2000 to 2500. The red, blue, violet, pink, and green solid lines denote the *OTEC3*, *OTEC5*, *OTEC7*, *OTEC10*, and *OTEC15* RCP8.5 scenarios, respectively

For the first 200 years of the simulations, power generation rates were limited by the OTEC production goals in all scenarios aside from *OTEC15* (Fig. 4a). Power output in *OTEC15* diminished below the power generation goal by 2150, despite remaining below the climate mitigation power demand limitation. This indicates that OTEC power generation was restricted by a paucity of suitable OTEC locations. Between 2150 and 2250, all subsequent OTEC scenarios became limited by climate mitigation power demand, and power production diminished to less than 1.5 TW for the remainder of the modelled period (Fig. 4a). OTEC scenarios with greater power outputs became limited by emissions before those with lower outputs. By 2500, OTEC scenarios produced a mean power of 0.6 TW (Fig. 4a).

It was assumed that OTEC replaced fossil fuel-intensive forms of energy and, therefore, OTEC implementation was associated with a reduction in carbon emissions (Fig. 4a). The magnitude of the emission reductions was directly proportional to the level of OTEC power generation. Emission reductions began in 2030 with the onset of OTEC power production, rose to peak values by 2100 and declined by 2250 when the climate mitigation power demand began to limit OTEC power generation. While the extension of RCP8.5 assumed stabilized CO_2_ concentrations after 2300, a small amount of diagnosed anthropogenic CO_2_ emissions remained. These emissions were the amount required to counteract the natural sinks of atmospheric CO_2_ to maintain the constant level of CO_2_ consistent with the RCP8.5 scenario without OTEC. These emissions were still replaced by OTEC, and therefore, emission reductions never fell to zero but rather reached annual average values of about 1 Pg of carbon by 2500 (Fig. 4a).

OTEC plant deployment broadly reflected trends in the level of OTEC power generation with a greater number of plants required as power output increased (Fig. 4b). At peak OTEC deployment, the total number of plants ranged from 14,000 (*OTEC3*) to 150,000 (*OTEC15*). One notable difference from power production trends is that, while the specified power production remained constant between 2100 and 2250, the number of OTEC plants required to meet the specified power output continued to rise. The need for an increased number of plants during this time is a product of the decrease in plant efficiency that occurs over time. This is particularly noticeable at higher power goals. As OTEC produces power, DOW is upwelled and released near the sea surface. This leads to a cooling of surface waters, which decreases the shallow-to-deep temperature gradient that powers OTEC, making the technology less efficient. Climate warming also contributes to a reduction of these gradients over the longer, term by decreasing the amount of cold deep water that is formed in polar regions. The ‘noise’ seen between the years 2150 and 2200 in the 15 TW scenario (Fig. 4) occurred due to an insufficient number of viable locations available to produce the power required to displace fossil-fuel-generated electricity given the RCP8.5 forcing scenario.

#### Carbon dioxide and temperature

**Fig. 5:**
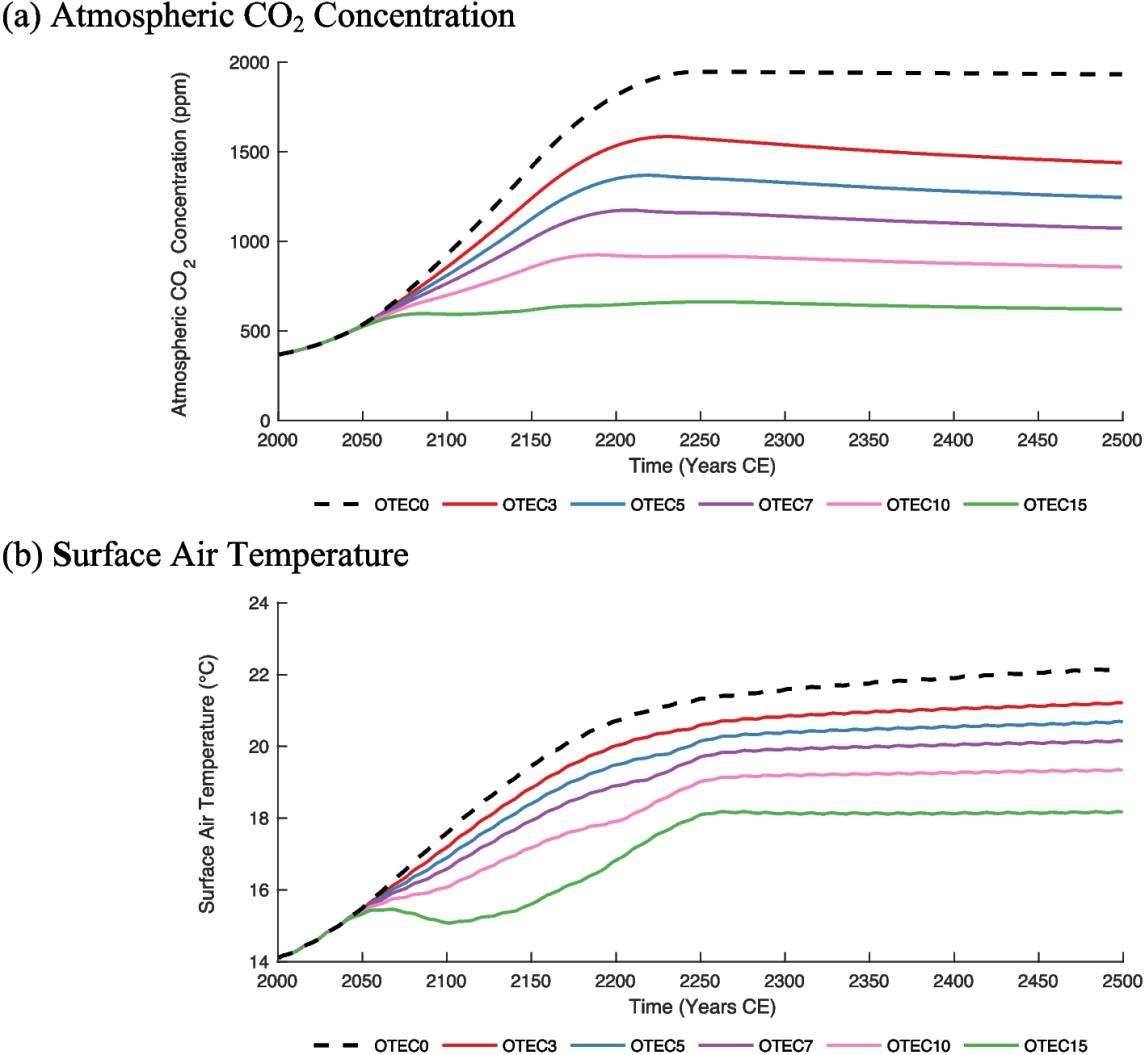
(**a**) Globally averaged atmospheric CO_2_ concentration in parts per million (ppm). (**b**) Globally averaged surface air temperature in ºC. The black dashed, red, blue, violet, pink, and green solid lines denote the *OTEC0, OTEC3*, *OTEC5*, *OTEC7*, *OTEC10*, and *OTEC15* RCP8.5 scenarios, respectively

Atmospheric CO_2_ concentrations in *OTEC3*, *OTEC5*, *OTEC7*, *OTEC10*, and *OTEC15* increase to varying extents over the first 200 years of the simulation (Fig. 5a) due to the high level of anthropogenic CO_2_ emissions present in the RCP8.5 scenario. After this period of increase, CO_2_ concentrations plateau and slowly decline. Since it is assumed that OTEC is only replacing more fossil-fuel-intensive energy systems, OTEC leads to CO_2_ emission reductions. As rates of power production increase, so do carbon emission reductions, as OTEC incrementally replaces more power production derived from fossil fuels. Post 2250, CO_2_ emissions in RCP8.5 are greatly reduced. Therefore, the emission reduction associated with OTEC is also decreased, as there are no longer significant emissions to be replaced. Consequently, atmospheric CO_2_ concentrations in all modelled scenarios remain relatively constant from 2250 onwards. In 2500, atmospheric CO_2_ concentrations range from 623 ppm (*OTEC15*) to 1930 ppm (*OTEC0*), depending on the level of OTEC power generation (Fig. 5a). By the year 2500, OTEC power generation resulted in cumulative emission reductions of 323 (*OTEC3*) to 981 (*OTEC15*) Pg of C relative to a control scenario without OTEC deployment. These reductions in carbon emissions are equivalent in magnitude to 36% to 111% of historical anthropogenic carbon emissions from 1750 to 2023 (Ritchie et al. 2017).

The overall effect of OTEC mixing on atmospheric CO_2_ is complex. OTEC mixing brings cool DOW to the surface, which increases CO_2_ solubility and oceanic CO_2_ uptake. DOW is rich in essential nutrients which increase biological productivity and the strength of the biological pump, which can again increase oceanic CO_2_ uptake. However, the upwelling of pCO_2_-rich DOW would increase atmospheric CO_2_ outgassing. Which processes dominate depends on the scenario and will vary in space and time. At peak OTEC power generation, in 2100, *OTEC10* experiences a 224.5 ppm decrease in atmospheric CO_2_ concentrations relative to the same year in *OTEC0*. If the emission reduction from OTEC power production is eliminated, in a similar OTEC10 scenario, CO_2_ decreases by only 23.1 ppm indicating that about 10% of the overall reduction in CO_2_ is attributable to DOW upwelling and the remaining 90% is caused by OTEC emissions reductions. During this time, OTEC-induced mixing enhances the flux of carbon from the atmosphere into the land and surface ocean. In a simulation similar to *OTEC10* but excluding emissions reductions, the land carbon pool experiences a 2.5% increase by 2100 relative to *OTEC0* to a global total of 1730 Pg of carbon. Total ocean carbon increased by 0.02% from *OTEC0* to a total of 37700 Pg of carbon in 2100. Total atmospheric carbon in year 2100 has a value of 1930 Pg, which is a 2.5% decrease from *OTEC0*. After OTEC is largely terminated, OTEC-induced mixing causes a slight reduction in the flux of carbon to the land and surface ocean and, by 2500, the land and ocean carbon pools have both decreased by 0.4% while total atmospheric carbon has risen by 0.12%. By this time, OTEC-induced mixing contributes less than 1% to the changes in atmospheric CO_2_ concentration and the remaining changes are a result of residual emissions reductions.

**Fig. 6:**
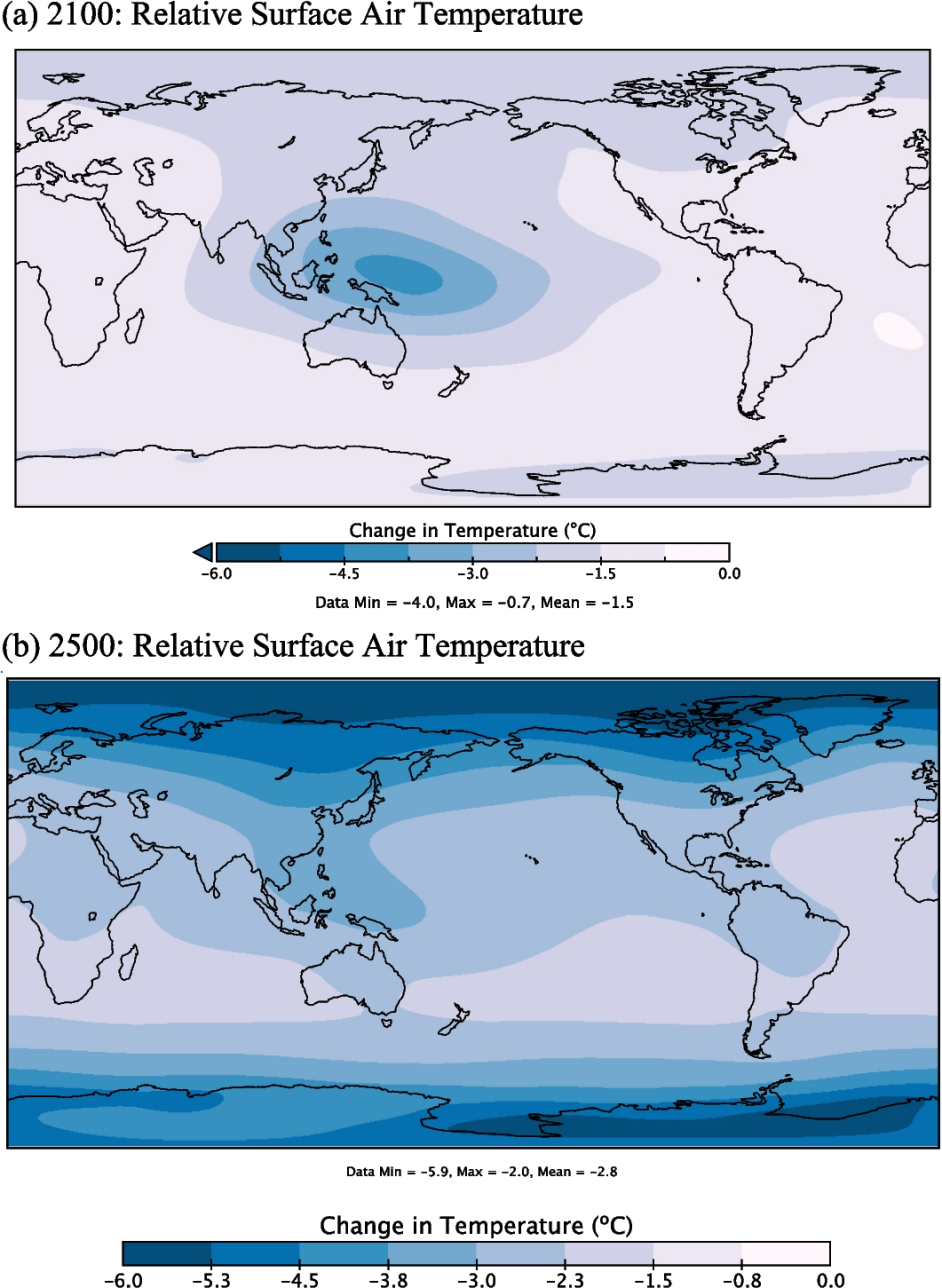
Change in surface air temperatures in ºC in 2100 (**a**) and 2500 (**b**) for the *OTEC10* RCP8.5 scenario relative to the same year in the *OTEC0* RCP8.5 scenario. Note that this relative cooling indicates less absolute warming

Although all modelled scenarios experience net surface air temperature increases over the modelled period, the implementation of OTEC leads to a suppression of this warming (Figs. 5b and 6). By 2500, relative to *OTEC0,* OTEC scenarios experience global surface air temperature decreases ranging from 0.95 ºC at the lowest level of OTEC power generation to 4.0 ºC at the highest. Much of the relative atmospheric cooling occurs before 2300, after which carbon emission reductions are greatly diminished, and CO_2_ concentrations are stabilized. Surface waters are also relatively cooled by OTEC mixing, promoting the exchange of heat at the ocean–atmosphere boundary, further contributing to the relative reduction of surface air temperatures. Since both these factors are amplified at greater rates of OTEC power production, the greatest reduction in surface air temperatures is seen in the *OTEC15* scenario (Fig. 5b).

While OTEC is largely operational (2030–2250), most of the relative surface air cooling occurs above areas with highly concentrated OTEC plant deployment and in polar regions (Fig. 6a). Enhanced relative cooling occurs at high latitudes as the result of a reduction in the ice-albedo feedbacks that caused increased warming in the *OTEC0* scenario (Fig. 6b). While scenarios with OTEC power generation experience ubiquitous cooling relative to *OTEC0*, the simulations do still experience increases in global average surface air temperatures relative to year 2000 values due to the considerable anthropogenic emissions included in RCP8.5 (Fig. 5b). However, these temperature increases do not occur ubiquitously. Relative to year 2000 values, most areas without OTEC deployment, notably the polar regions, experience warming, whereas areas with significant OTEC deployment experience a slight cooling with a maximum of 0.7 ºC by year 2100 in the *OTEC10* scenario.

Surface air temperature reductions from *OTEC0* are predominantly the product of two driving forces: (1) enhanced heat uptake to surface oceans in areas undergoing OTEC-induced mixing and (2) OTEC-related carbon emissions reductions. In 2100, OTEC power generation is at a maximum and changes to surface air temperature are primarily controlled by enhanced heat uptake from the atmosphere to the surface oceans. When considering both driving forces, global average surface air temperatures in *OTEC10* are lower by 1.5 ºC relative to *OTEC0*. When OTEC-related emissions reductions are not included, in a similar OTEC10 scenario, global surface air temperatures still decrease by 0.86 ºC. Thus, OTEC-induced mixing contributes just under 60% to the decrease in global surface air temperatures while OTEC-related emission reductions contribute just over 40% in 2100. Heat uptake is greatest in areas where OTEC-induced mixing is occurring, so the relative cooling is concentrated in the western equatorial Pacific with maximum values of − 4.0 ºC (Fig. 6a). By 2500, OTEC power generation rates have greatly diminished and persisting emissions reductions become the dominant driver of the relative surface cooling. *OTEC10* Global average surface air temperatures in 2500 are lower by 2.8 ºC relative to *OTEC0*, while simulations without emissions reductions warm 0.1 ºC above *OTEC0*. At year 2500, 104% of the global surface temperature reduction is from emission reductions while the residual effects of OTEC-induced mixing act in opposition and cause a 4% warming. While the rapid cooling from DOW upwelling has a large short-term effect it is only emissions displaced by OTEC power production that are important for long-term climate change mitigation.

## Conclusions

This study attempted to quantify the relationship between the rate and duration of OTEC power production and to explore the potential climate change mitigation potential associated with the production of renewable, continuously available energy with OTEC. Simulations indicate that ocean systems could briefly produce a vast amount of power (more than 35 TW), however, this level of power generation could be sustained for less than 30 years before areas with adequate shallow-to-deep temperature gradients are depleted. Once temperature gradients are depleted, power generation is reduced to values around 1 TW. Depending on the state of the climate, OTEC power generation levels of up to 5 to 10 TW may be sustainable on multi-century timescales before power generation collapses. Production rates less than 4.9 TW (year 2300 forcing) and 9.6 TW (year 2000 forcings) did not experience collapse over the observed 10,000-year period. These results suggest that, unlike previous estimates of sustainable OTEC power extraction rates, ocean reservoir depletion depends heavily on both the climate state and the rate and duration of power extraction. These power yields represent immense amounts of renewably sourced electricity, although the power generation process would not be without many potential environmental concerns.

OTEC presents many potential climate mitigation benefits. The generation of up to 5 to 10 TW of continuously available renewable energy for thousands of years is exceptionally valuable given the increasing need for more carbon–neutral forms of energy. OTEC is associated with relative atmospheric and oceanic cooling and emissions reductions which may help us reach goals set to limit warming and reduce the detrimental effects of climate change. Over 500 years, OTEC resulted in cumulative emission reductions of 323 (*OTEC3*) to 981 (*OTEC15*) Pg of carbon relative to a control scenario without OTEC deployment. The magnitude of these reductions represents 36% to 111% of 2023 cumulative anthropogenic carbon emissions since 1750 (Ritchie et al. 2017). While absolute temperatures continue to rise in all simulations, globally averaged surface atmosphere temperature increases in simulations with OTEC were smaller by 1.0 to 4.0 ºC relative to control values by 2500. Cooling trends are driven by the carbon emission reductions associated with the switch from a carbon-intensive form of energy to OTEC and OTEC-induced mixing of cold DOW to the sea surface. While OTEC is operational at a high level, OTEC-induced mixing contributes roughly 60% of the observed cooling while the remainder is the product of OTEC-related emission reductions. Once OTEC power production diminishes, nearly all cooling is a result of the sustained emissions reductions, and OTEC-induced mixing plays a much smaller role. While the relative carbon emission reductions and cooling described in this study would be immensely valuable for climate mitigation efforts, it is important to note that these impacts are not exclusive to OTEC and much of this mitigation could be achieved with other forms of renewable energy systems.

While earth models of intermediate complexity like the UVic ESCM are excellent tools for exploring the potential costs and benefits of OTEC, the UVic ESCM atmosphere is highly parameterized and excludes any potential cloud feedbacks. These simplifications limit the analysis of some important atmospheric phenomena and future environmental impacts will need to be investigated with even more comprehensive models. Despite these model limitations, OTEC appears to present many potential benefits both societally and environmentally. While financial barriers and engineering challenges remain, OTEC could potentially produce vast amounts of power and OTEC-related reductions in greenhouse gas emissions could be immensely valuable for mitigating the detrimental effects of climate change and aid in meeting goals set to limit warming.

## Data Availability

The code and data used in this study are available from the authors on request.
